# Seasonable Clothing

**Published:** 1883-08

**Authors:** 


// HALL'S JOURNAL OF HEALTH AND MISCELLANY.

MONTHLY.

E. H. GIBBS, A. M., M. D., EDITOR.

FOUNDED IN 1854.

### HALL'S JOURNAL OF HEALTH AND MISCELLANY.

### SEASONABLE CLOTHING.

THE preservation of health depends on maintaining a proper equilibrium of temperature for the body at all times. This is not attained exclusively by a seasonable regulation of the amount of clothing to be worn; but there are other and most important conditions to be considered. Clothing does not create warmth; it simply retains it after it has been created by the action of food, air and exercise on the wonderful and complicated machinery of the human system. But errors in dress have more to do with impairing the health than any other cause. The great object of clothing being to protect the body from sudden changes of heat and cold, it follows that in order to meet these changes, it is safest to be provided with an ample supply of garments of various weights and textures; and all things considered, it is wise in an economical point of view. Three suits of clothing will last three times as long as one suit; while the liability to disease is greatly lessened by systematic attention to appropriate dressing.

The effect of scanty clothing in cold weather is to allow the body to become chilled, thus causing the openings of the millions of pores in the skin to be more or less contracted and hindering the waste matters of the body from reaching the surface freely in the form of perspiration. A portion are thus thrown back into the system, and becoming clogged the new accumulations force them into the tissues of the body or into some vital organ, thus giving rise to disease in the part receiving the effete matter. This is why it is dangerous to keep up the full supply of food under these circumstances. It is adding fuel to the flame. The most rational way to avoid the dangers resulting from a cold is to eat nothing except a little soup or some other liquid food for 24 to 48 hours or until the appetite and the general health return. A laxative in mild cases will cut short the cold, but in severe cold a thorough cathartic—a dose of liver pills—taken on going to bed is the very best guarantee against the diseases that so frequently follow a cold, since a cold always induces a torpid condition of the liver.

Wearing too much clothing may induce diseases, which, though of an opposite character, are quite as destructive. The unnatural warmth of the body that results from too much clothing induces increased perspiration, whereby a large percentage of the nutritive particles that are required to feed the system and supply the natural waste of tissue are eliminated before they have finished their work ; hence the body becomes debilitated, the circulation is sluggish and the vital organs are soon reduced to a condition that renders them liable to permanent diseases from lack of power to resist them.

There are many persons who invariably have an attack of diarrhœa after every sudden change in the weather from heat to cold ; thus they escape the more serious consequences that are liable to overtake those not thus affected. The general observation of two simple rules would prolong thousands of useful lives :

First.—Always adapt your clothing to the changes of the weather.

Second.—Make entire personal comfort the test of health, as relates to the amount of clothing to be worn, or the quality or quantity of food to be eaten.

